# Identifying Hallmark Symptoms of Developmental Prosopagnosia for Non-Experts

**DOI:** 10.1038/s41598-018-20089-7

**Published:** 2018-01-26

**Authors:** Ebony Murray, Peter J. Hills, Rachel J. Bennetts, Sarah Bate

**Affiliations:** 1 Department of Psychology, Bournemouth University, Bournemouth, Dorset UK; 2 Department of Biological and Experimental Psychology, Queen Mary University of London, London, UK

## Abstract

Developmental prosopagnosia (DP) is characterised by a severe and relatively selective deficit in face recognition, in the absence of neurological injury. Because public and professional awareness of DP is low, many adults and children are not identified for formal testing. This may partly result from the lack of appropriate screening tools that can be used by non-experts in either professional or personal settings. To address this issue, the current study sought to (a) explore when DP can first be detected in oneself and another, and (b) identify a list of the condition’s everyday behavioural manifestations. Questionnaires and interviews were administered to large samples of adult DPs, their unaffected significant others, and parents of children with the condition; and data were analysed using inductive content analysis. It was found that DPs have limited insight into their difficulties, with most only achieving realisation in adulthood. Nevertheless, the DPs’ reflections on their childhood experiences, together with the parental responses, revealed specific indicators that can potentially be used to spot the condition in early childhood. These everyday hallmark symptoms may aid the detection of individuals who would benefit from objective testing, in oneself (in adults) or another person (for both adults and children).

## Introduction

Faces provide information about one’s gender, age, ethnicity, emotional state, and perhaps most importantly, they identify the owner. Thus, the ability to recognise an individual just by looking at their face is crucial for human social interaction. Prosopagnosia is a cognitive condition characterised by a relatively selective impairment in face recognition^1^. The disorder can be acquired (typically affecting occipitotemporal regions^2,3^) or developmental in nature, with the latter also referred to as “congenital” or “hereditary” prosopagnosia^4–6^. The condition occurs in the absence of any neurological damage, socio-emotional dysfunction or lower-level visual deficits^4^, and may affect 2–2.5% of the adult population^7^ and 1.2–4% of those in middle childhood^8^ (although note that, by definition, the lower end of a normal distribution would encompass 2.5% of the population and therefore, prevalence rates reflecting this may be a statistical artefact^9^).

In the last 20 years, individuals with DP have been used to make theoretical inferences about the development and functioning of the cognitive and neural architecture of the typical and impaired face recognition system (e.g. refs^10–14^). Given some individuals also report moderate-to-severe psychosocial consequences of the condition^15,16^, there has been increasing interest in the accurate diagnosis of DP via objective testing. Many researchers diagnose the condition using a combination of the Cambridge Face Memory Test (CFMT^17^) and the Cambridge Face Perception Test (CFPT^18^) - regarded as the leading objective tests of face recognition - and a famous faces test (e.g. refs^19–23^). Participants are thought to meet the diagnostic criteria for DP when their scores are considered together, and in many cases, this will mean that DP is determined when individuals score atypically on at least two of these three measures. Often to meet diagnostic criteria, impairment is evident on the two face *recognition* tasks as not all individuals with DP show impairments on the CFPT (e.g. refs^24–26^). Finally, most researchers agree that anecdotal evidence of everyday difficulties with face recognition is required to support a DP diagnosis^27,28^. Existing reports have mostly relied upon the individual to describe incidents of everyday failures of face recognition (e.g. refs^20,29^) although some researchers have developed more formal questionnaires or structured interviews for this purpose (e.g. refs^30,31^).

However, a more fundamental identification of the everyday behavioural traits that are associated with DP is an important issue, irrespective of any debates surrounding the formal diagnosis of the condition. Indeed, the objective diagnosis of DP typically requires an individual to initially self-refer (or be referred by a guardian or significant other) to a laboratory for screening, yet this process requires that person to have some awareness of their face recognition difficulties relative to unaffected others. Although some self-report tools have been shown to successfully identify at least some candidates for DP^32^, many anecdotal reports suggest a lack of awareness into the severity of one’s face recognition difficulties^33,34^. Further, much work examining the typical population indicates that people have limited insight into their own abilities (e.g. ref.^35^) and specifically into their face recognition skills, reporting only weak-to-moderate correlations between subjective ratings and scores on objective tests (e.g. refs^36–38^).

Unlike those with acquired prosopagnosia, those with DP have no point of comparison nor experience an abrupt loss of their face recognition skills: many individuals tested in our laboratory did not become aware of their difficulties until mid or even late adulthood (see also^33,34^). This is likely to be due to a combination of reasons. For instance, many people with prosopagnosia can identify people via voice, gait and general appearance and manner^15^. Face recognition difficulties have also been reported to be highly heritable (e.g. refs^39,40^) and individuals may be comparing their abilities to family members who are equally poor at recognising faces. Subsequently, these individuals may not become aware of their difficulties for a long period of time. Additionally, some people with DP devise their own strategies to recognise others and cope relatively well with their difficulties^33^. This may conceal the condition from other people, or even falsely indicate to oneself, that they are able to recognise others in the same manner as most others in the general population.

If an unaffected person is to recognise the traits of DP in others (as would typically be required to identify the condition in children), they must first know that the condition exists and have an understanding of its behavioural manifestation on an everyday level. Yet, public awareness of DP is still relatively low, and, although there has been a surge of research examining DP in adulthood, relatively little has been carried out with children. Many parents and educational professionals have therefore never heard of the condition. Some reports suggest that this lack of awareness can lead to misdiagnoses of alternative developmental disorders, such as anxiety disorders or attention deficit disorder^41^, or pervasive developmental disorder and autism spectrum disorder (ASD)^42^. This means that children with DP are not receiving suitable support, and in some cases, will use resources which may be better spent elsewhere.

The general public, professionals and researchers would therefore benefit from the identification of a list of traits that are typically associated with the condition. The few existing attempts to identify such symptoms of DP have mainly been drawn from informal discussions with those living with the condition and/or anecdotal reports in the available literature (e.g. ref.^30^). Formal discussions with DPs have been documented (e.g. refs^15,16^), but such work has primarily focused on the psychosocial consequences of the condition. Moreover, much of the qualitative work in this area also employs small sample sizes, or even independent case studies (e.g. refs^31,33,41^). In addition, to our knowledge no study has investigated the indicators of DP that could exist from the point of view of a person without the condition and, thus, how DP could be identified by others (but see ref.^16^ for coping, social and psychosocial adjustment in children with DP, from both children’s and parents’ viewpoints). Importantly, provision of a means for others to detect DP in an adult or a child may not only overcome the unreliability of self-insight into face recognition skills, but may decrease the likelihood of misdiagnosis and increase the specificity of the support they receive via referral to the appropriate outlet for more detailed objective testing.

The current study addressed these issues using questionnaires and semi-structured interviews that were used to explore the key indicators of DP. Data were collected from large samples of adults who meet the diagnostic criteria for the condition, their unaffected significant others, and parents of children with DP. Combining these data, the current paper addresses three main research aims. First, we investigated whether DPs have insight into their difficulties, and at what age insight is gained. The implications this has for subjective measures of face recognition skills are discussed. Secondly, we explore the age at which DP can potentially be detected by others. Finally, we identify sixteen hallmark symptoms for DP which can aid the detection of DP in oneself (in adulthood) and another person (in childhood and adulthood).

## Method

### Participants

All participants in this study completed a questionnaire about DP and some subsequently opted to participate in an interview. Fifty individuals with DP (mean age = 53.0 years, SD = 13.0; 17 male) completed the questionnaire and 23 (mean age = 53.9 years, SD = 13.3; eight male) went on to complete the interview. Twenty six unimpaired significant others (SOs; mean age = 52.6 years, SD = 13.0; 12 male) of the adults with DP also completed the questionnaire and seven (mean age = 62.1 years, SD = 11.3; one male) went on to complete the interview. Three parents (mean age = 43.5, SD = 7.5; one male) of children with DP (mean age = 9.0 years, SD = 4.5; three male) filled in the questionnaire and two also participated in the interview (parents: mean age = 45.0 years, SD = 9.9; one male; children: mean age = 5.0, SD = 7.1, two male). Further details of the participants who completed each component of the study (e.g. their objective test scores) is provided in the Supplementary Information. Informed consent was obtained from all participants included in this study. They participated on a voluntary basis. The study was carried out in accordance with Bournemouth University Research Ethics Guidelines and was approved by the Bournemouth University Research Ethics Committee.

### Participants with DP

All DP participants had previously contacted the research team reporting difficulties with face recognition. All had normal (or corrected to normal) vision, no learning disability or any other neurodevelopmental disorder (including ASD), and no known history of neurological damage or psychiatric illness. Thus, their difficulties were regarded as developmental in origin. Following existing protocols (e.g. ref.^7^) all participants were screened using the CFMT^17^ and the CFPT^18^. Some also completed a famous faces test^20^.

In the CFMT, participants are required to learn six new male faces and are tested using a three-alternative forced choice format. The test is made up of four sections which increase in difficulty as the test progresses: practice, introduction/same images, novel images, and novel images with noise. Responses in the practice section are not recorded. In the latter three stages there are a total of 72 recorded trials. The measure is the number of correct trials. Thus, the maximum score on the CFMT is 72; chance is 24.

Any deficits identified using the CFMT may result from impairments restricted to memory. However, they may arise from impairments earlier on in facial identity processing. To examine this, the CFPT is a measure of face perception which does not rely on memory. The CFPT is a computerised sorting task whereby participants are required to organise six faces according to their similarity to a target face. The six faces which require organising were created by morphing the target face with six other individuals. The proportion of the morph coming from the target image is varied in each face which requires sorting: 88%, 76%, 64%, 52%, 40% and 28%. Eight different sorts were created and each is presented upright once and inverted once. In sum, there are a total of 16 trials. Scores on the CFPT are computed by totalling the deviations from the correct position of each face and so the higher the score the poorer the performance. Totals for the eight upright and eight inverted trials are calculated separately. Existing diagnostic protocols (e.g. ref.^19,20^) only examine performance on the upright trials, so we only report those in this paper.

To assess familiar face recognition, we used a famous faces test that has previously been used in our published work^20,43^. Participants were presented with 60 famous faces, one at a time, on a computer screen. Each face was cropped so that little extrafacial information was visible. Participants were asked to identify the person by either naming them or by providing some uniquely identifying autobiographical information about that person. They were able to take as long as they needed to provide such information. Participants were provided with the name of any face they failed to identify and asked if they had substantial exposure to that person in the past. If the participant did not feel that they were familiar with that celebrity, that trial was eliminated from their final score. Scores are therefore reported as percentages.

Individuals were considered to meet the current diagnostic criteria for DP if they scored atypically on at least two out of three of the above measures. Some participants had not completed the famous face test, and these individuals therefore had to be impaired on both the CFMT and CFPT for inclusion in this study. Following existing protocols (e.g. refs^7,17^), atypical scores were deemed to be those that fell more than 2 standard deviations below the control mean. For the CFMT, these are scores equal to or less than 42. For the CFPT, these are scores which are equal to or more than 61, and for the famous faces test, these are scores which are approximately 50% and below (please see each accompanying publication for further details of the norms and atypical scores). A full set of scores can be found in   the Supplementary Information.

### Significant other participants

When the DP participants volunteered to participate they were also asked if they had an unaffected SO who was happy to participate in the study. Twenty six SOs volunteered to take part, 24 of whom were long-term romantic partners of the DPs. Two SOs were family members (one daughter, one mother).

### Parental participants

All parents had previously contacted the research team as they were concerned about their child’s ability to recognise faces. Children had normal (or corrected to normal) vision, no diagnosis of any other neurodevelopmental disorder or learning disability, and no known history of neurological damage or psychiatric illness. Thus, their difficulties were regarded as developmental in origin. All children attended a screening session at Bournemouth University and, following existing protocols^8,44^ were identified as meeting the diagnostic criteria for DP if they performed atypically on the following tests.

The CFMT-Kids^44^ is a face memory test, matched in format to the original CFMT. Child faces rather than adult faces are used as stimuli. Children aged eight and younger complete a short version in which the child only learns four faces. The short version has a total of 48 trials (12 trials in the learning stage; 20 in the test stage with novel viewpoints; 16 in the test phase with noise overlaid). Children over eight years of age complete the full version of the task, which requires the learning of six faces. Similarly to the adult test, the full version has a total of 72 trials (18 trials in the learning stage; 30 in the test stage with novel viewpoints; 24 in the test phase with noise overlaid). Scores were converted to percentages to facilitate comparisons across the two versions of the test.

Similar to the adult version of the CFMT, any deficits identified using the CFMT-Kids may result from impairments restricted to memory. Further, face perception tasks have been proposed as a better choice of diagnostic test in child populations^45^. Thus, a face perception test was also administered^8^: a three-alternative forced choice simultaneous matching task consisting of 30 trials. A target stimulus is shown at the top of the screen, along with three test stimuli at the bottom. In order to avoid simple image matching, target and test stimuli differ in viewpoint and/or lighting conditions. On each trial, participants are asked to choose which of the test stimuli is the same identity as the target stimulus and to respond using the 1, 2, and 3 keys on the keyboard. The stimuli remain on screen until a response is made.

Children were considered to meet the current diagnostic criteria for DP if they scored atypically on both of the above measures. Following existing protocols atypical scores were deemed to be those that fell more than two standard deviations below the control mean for that child’s age group (norms and atypical scores are detailed further in the accompanying publications). A full set of scores can be found in the Supplementary Information.

## Materials and Procedure

### Questionnaires

Questionnaires consisted of 7 main questions (see Supplementary Information). Participants were able to complete the questionnaire in a number of ways. Most were sent the questionnaire via email, and returned an electronic copy of their responses in the same manner. Alternatively, they could complete the questionnaire using an online survey platform. Four participants requested a paper copy of the questionnaire, which was sent and returned via the post. Two participants completed the questionnaire on paper when they visited the university to take part in a different research project.

Questions were mostly open in nature and, given that our aim was to identify ways in which DP can be detected, were designed to encourage participants to draw upon experiences which highlighted their face recognition difficulties. For instance, participants were asked the age at which they felt their difficulties became apparent, the severity of their difficulties, and what they would consider to be hallmark symptoms of the condition. Adaptations of these questionnaires were created for SOs and for parents (see Supplementary Information), enquiring about their SO’s or child’s face recognition difficulties. All members of the research team checked questionnaires and questions for comprehensibility. The lead author was available to answer or clarify any issues raised by the participants via e-mail or telephone prior to and during participation. However, no participants required such assistance. We estimated completion time to be approximately 15 minutes but made it clear that due to the nature of the questionnaire, this would very much vary between participants.

### Interviews

Interviews were of a semi-structured format. Set questions were developed and created to complement and extend upon the questionnaire items. There were a total of three main questions for DP participants, three for SOs and five for parents (see Supplementary Information). In addition, any ambiguous or under-developed answers that had been provided in the questionnaires were clarified during the interview. Subtle prompts, such as “would you mind expanding on that answer?” or “what makes you say that?” were provided by the interviewer when expansion was required.

Interviews were carried out by the first author who had either met participants in the past and/or had communicated with the participants via e-mail prior to the interview. Thus, participants had been introduced to and were anticipated to feel comfortable with the interviewer. All participants had discussed the interview process with the first author and fully understood this process prior to the interviews. They were also fully aware that interviews were going to be audio recorded. The first author was also available to answer any questions form the participants at any time prior, during, or after the interviews. All interviews were audio recorded using a ReTell 156 Telephone Handset Call Recording Connector and an Olympus VN-731 PC (2 GB) recorder. These interviews were transcribed by a third party and then checked for accuracy by the first author. Due to the nature of the interviews, their length substantially varied from person to person. For DPs, the interviews ranged from 10.0 minutes to 59.3 minutes, presenting a mean length of 18.1 minutes; for SOs, interviews ranged from 10.1 minutes to 50.1 minutes, presenting a mean length of 19.6 minutes; for parents, interviews ranged from 11.3 minutes to 50.2 minutes, presenting a mean length of 36.2 minutes.

## Analyses

### Qualitative Analyses

Qualitative data were analysed using inductive Content Analysis (CA). CA is a method of analysing written, verbal and/or visual communication messages and is a systematic and objective means of describing and quantifying phenomena^46^. Very simply, CA involves exploration of the data in order to note how many times a category appears. CA is sometimes treated as similar to thematic approaches (e.g. ref.^47^), as used by Yardley and colleagues^15^, and Dalrymple and colleagues^16^. However, CA tends to focus at a more micro level, provides frequency counts, and uses words or phrases as the unit of analysis. Comparatively, the unit of analysis tends to be more than a word or phrase in thematic analysis^48^. CA was the chosen method for analysis here as it is an unobtrusive method which accommodates large amounts of data easily^49^. Therefore, the number of participants and amount of qualitative data which required analysing influenced the selection of this technique.

CA has been a fairly common analysis method used in a number of subject areas (e.g. nursing, media and dementia) although to our knowledge, it has not been used as a data analysis method within the DP literature. Inductive CA was conducted on the data following the guidelines laid out by Elo and Kyngas^50^. Following these guidelines, data were prepared and the selected unit of analysis was phrases (rather than single words, full sentences, or themes). This meant that the unit of analysis would not be too narrow to result in fragmentation or to lose context of the responses. The data were then open coded, and codes were counted (i.e. how many times did that code appear through the data) before being grouped into higher order headings. The aim of grouping data was to reduce the number of categories by collapsing those that are similar into broader higher order categories. The number of times a category appeared was calculated by summing all of the codes’ frequencies which fell into this category. Abstraction then occurred; this meant that a general description of the research topic was formulated through the generation and naming of categories. This process was completed by the first author and the second author followed-up on the whole analysis process and categorisation.

### Data Availability

The datasets generated and analysed during the current study are available from the corresponding author on reasonable request.

## Results

Categories that emerged from the qualitative data analysis are initially presented. Results which directly refer to the three main research aims are then offered and elaborated.

### Content Analysis

Participant responses to questions across both the questionnaires and interviews described a variety of different experiences of living with DP and ways in which the condition might be detected in oneself and others. Qualitative data were analysed for each group (i.e. for DPs, SOs and parents) and different categories were derived for each. Occurrences of categories and definitions of these categories are described in more detail below.

### DP

After conducting inductive CA on the data from the DP participants, a total of five categories were generated (see Table 1). Descriptions and examples of these categories are presented in Table 2. Table 1 shows the number of individuals who discussed that category at any one time during the questionnaire and/or interview; these are referred to as ‘individual mentions’. The total number of mentions is also provided. Due to the nature of the questionnaire and interview, participants were able to discuss a category more than once in their responses. For example, one participant mentioned that they rely on someone’s gait and walk in order to recognise them, before later mentioning that they once failed to recognise a close family member because they were wearing a hat (and therefore their hair was covered); both of these were generalised and contributed to the category “Reliance on Extrafacial Information”.

**Table 1:** Content Analysis Table for DPs.

Category	Number of individuals who mentioned the category	‘Positive’ mentions^a^	‘Negative’ mentions^a^	*Number of mentions overall*
Reliance on Extrafacial Information	45 (90%)	45	0	*252*
Group and Social Contexts	45 (90%)	45	0	*212*
Importance of Context	43 (86%)	43	0	*81*
Insight and Implications for Self-Referral	39 (78%)	10	29	*144*
Alternative Explanations	31 (62%)	31	0	*83*

^a^Positive and negative mentions offer more insight into the categories. For example, DPs who believe that those with the condition do have insight into their difficulties are considered a positive mention whereas those who believe that DPs do not have insight into their difficulties are considered a negative mention. This is elaborated within the Discussion. The categories revealed by the DP data and the number of individuals who discussed that category.

**Table 2:** An Elaboration of Categories from DP Responses.

Category	Description	Quotes
Reliance on Extrafacial Information	DPs claimed that recognising an individual is made easier if an extrafacial cue is available for them to rely on. This included, but was not limited to, hairstyles and colour, gait, accessories and voice.	“People who have a particular characteristic – voice, size and shape, etc. are much easier [to recognise]” [DPF68] “More ‘individually looking’ people are easy. By that I mean wear unusual or distinctive clothing, characteristics e.g. very neat in dress, particular facial things e.g. type of moustache” [DPM72] “People with distinctive body shapes or very bright hair or unusual glasses or beards are easier [to recognise]. Gait is often a giveaway”. [DPF52]
Group and Social Contexts	DPs discussed experiences in which their face recognition difficulties impact, or has impacted, their behaviour in social contexts. This included, but was not limited to, avoiding introductions and names, avoiding certain social situations, appearing to be shy, or mixing people up with potentially embarrassing outcomes. These appeared to be worse for many DPs when in group settings and presented with a number of faces at one time. This included at social gatherings and parties, or when watching television or film.	“I never introduce myself to people or introduce people to each other or say anything that would only be appropriate for particular individuals.” [DPF49] “I would always call them ‘Sweetheart’ or ‘Honey’ etc. because I couldn’t be quite sure the child in front of me was the one I thought it was” [DPF54] “Trouble keeping characters or plots straight while watching movies (or for children, excessive questions about the movie)” [DPF27] “In a nursery full of babies I had no idea which was mine. As my children grew, pick up time at school was a nightmare if they didn’t see me first.” [DPF52] “I sometimes don’t even ‘see’ my own fiancé from a distance if we go to different parts of the supermarket” [DPF39]
Importance of Context	DPs highlighted the fact that their difficulties are more prominent when they are required to recognise an individual out of their normal context.	“I am sometimes still completely flummoxed when people are out of context, and this can happen with regular acquaintances. ” [DPM53] “[Difficulties are more prominent when] I’m out and about, and encountering people where they are totally unexpected” [DPF54] “Recognition is harder in supermarkets when people stop and say hello, or passing by on the street.” [DPF53]
Insight and Implications for Self-Referral	DPs discussed how people with the condition do not have insight into their difficulties (negative mentions), which causes implications for self-referral and potential diagnosis. It is worth noting here that 6 DPs (12%) stated insight is only gained when a point of comparison is available, and 15 DPs (30%) noted that insight is only gained when made aware that it is a recognised condition.	“It is only other people telling you that makes you realise the problem. I think it’s a bit like short-sightedness, you only realise you have that when someone expresses surprise that you can’t read a sign” [DPM51] “Why would it occur to someone that they see or remember other people differently? I think, like me, they probably mostly assume they are just like everyone else in the way they see and remember people but that their skills in this area are weaker” [DPF52] “You might not know what it is or what it is called, but as humans are very social beings, I think even a small child would know if they felt ‘different’ to their friends” [DPF54]
Alternative Explanations	DPs reported that they, or others, have attributed their difficulties to something else. This included, but is not limited to, thinking one was bad with names (not faces), thinking they were being rude or lazy, or believing their difficulties were due to not paying attention.	“I thought that I was just rubbish, wasn’t trying hard enough and was lazy” [DPF29] “I thought it was a social skill I hadn’t learnt due to short-sightedness as child” [DPF48] “I was seen as shy/formal/reserved as a young adult, but I am fairly relaxed and extroverted” [DPM63]

An elaboration of the categories generated through CA on DP responses. Suitable descriptions and quotations which illustrate these categories are presented.

It is important to note here that questionnaire and interview data were analysed together. Fifty DPs took part in the questionnaires to create five categories. Twenty three DPs participated in the interviews and these data contributed to these five categories.

### Significant Others

After conducting inductive CA on the data from the SO participant group, a total of three categories were generated (see Table 3). Descriptions and examples of these categories are provided in Table 4. Table 3 shows the number of ‘individual mentions’ and the total number of mentions. Similar to the analysis of the DP data, questionnaire and interview data were analysed together here. Twenty seven SOs took part in the questionnaires to create three categories. Seven SOs participated in the interviews and these data contributed to these three categories.

**Table 3:** Content Analysis Table for SOs.

Category	Number of individuals who mentioned the category	‘Positive’ mentions^a^	‘Negative’ mentions^a^	*Number of mentions overall*
Group and Social Contexts	22 (84.62%)	21	1	96
Importance of Context	18 (69.23%)	18	0	40
Reliance on Extrafacial Information	12 (46.15%)	12	0	38

^a^Positive and negative mentions offer more insight into the categories. For example, SOs who said that DP does impact their behaviour is social contexts and in groups are considered a positive mention whereas those who said that their significant other’s DP does not impact their behaviour is social contexts and in groups are considered a negative mention This is elaborated within the Discussion. The categories revealed by the SO data and the number of individuals who discussed that category.

**Table 4:** An Elaboration of Categories from SO Responses.

Category	Description	Quotes
Group and Social Contexts	SOs reported that their significant others’ difficulties had an impact on their behaviour in social contexts. This included, but was not limited to, avoiding social gatherings, coming across as disinterested or rude, and/or never introducing people or themselves to others. These were reported to be worse for many of the DPs when in group settings and presented with a number of faces at one time. This included at social gatherings and parties, or when watching television or film.	“When he meets people in the street he doesn’t introduce me” [SOF51] “Avoidant behaviour, does not engage on an emotional level, avoids physical contact and intimacy, and can come across as disinterested in others” [SOF46] “[she struggles to find me] when I am in a crowd” [SOM56] “Regularly identifies a face on TV as someone else and is 100% sure they have identified them correctly, but is wrong” [SOM54]
Importance of Context	SOs highlighted that seeing a person out of context made their significant others’ difficulties more prominent.	“I was aware that she seemed “poor” at recognising actors in different contexts” [SOM53] “He also has problems when meeting people out of context (e.g. a not so well known, but often seen neighbour, away from our ‘home streets’).” [SOF62] “It seems that the main difficulty is recognising people out of context. For example, when someone is known in the office or church, they are not immediately recognised in the street.” [SOM63]
Reliance on Extrafacial Information	SOs discussed the fact that their significant other often relies on extrafacial information to recognise a person, and that recognition fails when such information changes (including, but not limited to, one’s hairstyle, clothing or accessories).	“In the past, distinctive hair styles seem a valuable cue for recognition” [SOM53] “He cheered for the wrong person in a sports match because they had the same colour hair as me” [SOF19]

An elaboration of the categories generated through CA on SO responses. Suitable descriptions and quotations which illustrate these categories are presented.

All three categories derived from the SO data replicated those revealed within the DP data. Similar percentages of participants discussed these categories with only one major difference. “Reliance on Extrafacial Information” was discussed by 90% of the DPs compared to 46% of SOs. Overall, however, the categories and patterns within the categories were similar between the groups. For example, the categories “Group and Social Contexts” and “Importance of Context” was discussed by similar proportions of participants, and neither groups discussed these categories negatively (i.e. zero negative mentions).

### Parents

After conducting inductive CA on the data from the parent participant group, a total of five categories were generated (see Table 5). Descriptions and examples of these categories are provided in Table 6. Table 5 shows the number of ‘individual mentions’ and the total number of mentions.

**Table 5:** Content Analysis Table for Parents.

Category	Number of individuals who mentioned the category	*Number of mentions overall*
Social Interactions	3 (100%)	42
Reliance on Extrafacial Information	3 (100%)	36
Groups, Photography and Media	3 (100%)	16
Misdiagnosis and Professional Input	3 (100%)	12
Importance of Context	2 (66.6%)	5

The categories revealed by the parental data and the number of individuals who discussed that category.

**Table 6:** An Elaboration of Categories from Parent Responses.

Category	Description	Quotes
Social Interactions	All parents reported that their child’s DP impacted their social interactions, behaviour and mood. This included, but was not limited to, a poor ability to read one’s expression and body language, social skills developing later than average, and having few friends.	“He treated every child the same, with no preference for a particular child even though I knew that he knew some of those children extremely well” [PM15] “Taking time to warm up when meeting familiar people” [PM5] “I know absolutely he doesn’t want to go to the school prom which will be next year… They’re not with their school bags. And they do their hair differently and they’re all in penguin suits” [PM15]
Reliance on Extrafacial Information	All parents reported that their child uses extrafacial cues to recognise someone and often misrecognises individuals when these cues change or are shared. Such information includes, but is not limited to, hairstyles, voices, and facial hair.	“Voice, clothes and hairstyles also help” [PM6] “She has been unsure if I am who I am when I’ve had my hair cut and straightened” [PM6] “…would regularly identify people by their coats” [PM5]
Groups, Photography and Media	All parents discussed that their child’s difficulties were more prominent when presented with groups of people or faces. This included in the real world in real-time, but also included the child not being able to recognise someone (or themselves) in a photograph when asked, and an inability to follow films or plays.	“Asking about the identity of familiar people in photographs” [PM5] “Large crowds such as a London railway station or street markets are difficult” [PM15] “[Difficulties are more prominent in] places where there are large groups of people in a one place” [PM6] “It became obvious that he found it difficult to find me in the playground at the end of school” [PM15]
Misdiagnosis and Professional Input	Parents reported that alternative explanations for their child’s difficulties had been considered, some with professionals. These included a diagnosis of Autistic Spectrum Disorder, dyspraxia, and general underdeveloped social skills.	“No [medical professional] we spoke to had any experience. They were certainly open to finding out more about it but it wasn’t something that they could help with” [PM5] “…his nursery school when we first told them that we thought that there was a problem with facial recognition, they told us that they [had considered] him being on the autistic spectrum” [PM5]
Importance of Context	All parents stated that their child’s difficulties become more prominent when that person is out of context, and/or that relying too heavily on contextual cues results in misidentification.	“Relying too heavily on context/location and consequently misrecognising people” [PM6] “He would ask who familiar people are, who we have encountered out of context.” [PM5]

An elaboration of the categories generated through CA on SO responses. Suitable descriptions and quotations which illustrate these categories are presented.

Questionnaire and interview data were, again, analysed together. Three parents took part in the questionnaires to create four categories. Two of these parents went on to participate in the interviews and the interview data contributed to these four categories. The category “Misdiagnosis and Professional Input” was mentioned in the questionnaires by all parents. However, it was the opportunity for the parents to elaborate upon this within the interviews, which led to this being discussed enough to become a category in itself. Hence, with the data combined, five categories were revealed in total.

All five of the categories revealed within the parent data replicated those identified within the DP data. The three categories revealed by SO responses were replicated here. In sum, the categories referring to context, extrafacial information, and social and group contexts (including media), were discussed by all three groups of participants. Again, these categories were discussed by a similar proportion of participants within the DP and parent groups (e.g. 90% of DPs and 100% of parents discussed the role that extrafacial information plays in theirs, or their child’s, recognition abilities). Alternative explanations/misdiagnosis was a theme discussed by both the DPs and parents, but not by SOs.

### Subjective Measures of Face Recognition Skills

As seen in Tables 1 and 2, 39 of the 50 DPs discussed the category “Insight and Implications for Self-Referral”. Of these 39 individuals, only 10 believed that DPs in general (i.e. not necessarily talking about themselves) have insight into their own (in)abilities. For example, two DPs described that even as a small child they knew they were ‘different’ to their peers, even if they were unaware at the time that this was DP. Mostly, however, DPs believed that they do not have insight into their own face recognition difficulties and many of these individuals offered reasons why this is the case. Fifteen DPs stated that gaining insight into their abilities only occurred when they discovered what DP is (e.g. when they read an article in the media). Six DPs suggested that insight is only gained when there is an available point of comparison. Another factor which was discussed multiple times was that insight was gained after a move to university or a new workplace where a large number of people were introduced at once. Further, four of the DPs are or have been a teacher, and reported that this career helped them gain insight and identify the extent of their difficulties.

**Figure 1:**
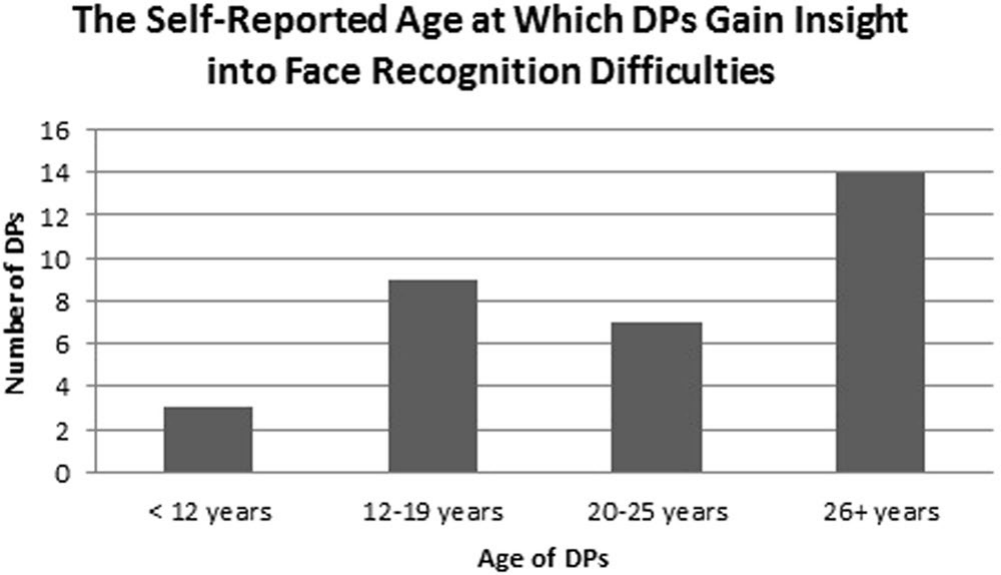
The self-reported age at which DPs gain insight into their face recognition difficulties.

Due to the variety of factors which appear to impact one’s ability to gain insight into face recognition difficulties, it is unsurprising that the proposed age at which insight is gained varied amongst responses. However, reported ages were suitably grouped (e.g. participants stated they gained insight in primary school, which is between the age of 7 and 11), and Fig. 1 displays the self-reported ages at which the DPs believed they gained insight into their difficulties. Note that data are presented for 33 DPs. Only 39 of the DPs discussed their category, and 6 of these individuals did not specify an age at which they gained insight. For example, four participants stated that gaining insight into one’s face recognition difficulties would depend on the severity of these difficulties. Thus, they contributed to this category but did not state an age at which they gained insight.

### Age of Potential Detection

As discussed above, many of the DPs realised the extent of their difficulties and gained insight into their (in)abilities in adulthood. However, with hindsight and when describing early experiences which demonstrate their difficulties, DPs varied in the age at which these experiences occurred. Forty two of the DPs discussed this in detail and were specific enough in their estimations to have these data analysed. Table 7 outlines the ages that these DPs first recalled a specific experience associated with their face recognition difficulties. Due to the qualitative nature of the present study, age brackets are created from the data (e.g. participants stated they gained insight in primary school, which is between the age of 7 and 11) and some ages are not reported (i.e. no DP participant believed their first experience occurred at 12 or 13 years of age).

**Table 7:** Earliest Experiences of DP.

Age at which DPs reported their earliest experiences	Number of participants
2–3 years (nursery)	4
4–6 years (starting school)	6
7–11 years (mid-late primary school)	16
14–16 years (teenage years)	8
18–20 years	2
Late 20 s	3
30 years	2

The age at which DPs first experienced their face recognition difficulties. Please note that only 42 DPs stated clear age brackets when asked how old they were when they first experienced their face recognition difficulties and, consequently, only 42 of our DPs’ data are presented here.

DP is typically defined as a life-long impairment in face recognition. One would then hypothesise that all of our DPs would recall an experience from childhood. However, a number of factors could influence the likelihood of this occurring. For instance, individuals may be unable to recall one specific event from such early childhood, or they may have had ‘coping’ mechanisms in early childhood. For example, one DP stated “when I was at small schools in 1950s and 60 s, pupils sat at the same place for most lessons, and twice daily register calls probably helped to ‘fix’ names and faces”. Another DP detailed that “the problem only really arises when I see people out of context, and at school you expect to see the same people in the same places”. We’d also expect some recall bias and memory bias when asking participants to recall events from their past. For instance, if the earliest experience of one’s face recognition difficulties elicited a severe negative emotional response, this memory would fade quicker and, consequently, not be easily recalled (the Fading Affect Bias^51^). Consequently, these factors mean that the age ranges presented here are fairly conservative estimates.

As seen in Table 7, 62% of DPs reported that they first experienced their difficulties in childhood (i.e. before the age of 11 years). Further, all 50 DPs stated in the questionnaire whether or not, with hindsight, they had difficulties recognising faces since childhood. 32 DPs (64%) could say categorically that they believed this to be the case. Two of the three parents reported that they suspected DP when their child was 4 years old, and the final parent suspected DP when their child was 5 years old. Because the majority of SOs were romantic partners and met their DP significant other in adulthood, it is impossible to say whether SOs would have or could have detected DP early on. Overall, however, the findings suggest that a reasonable proportion of DP cases can be identified in early childhood, and the majority can be detected by mid-childhood.

### Hallmark Symptoms

Across all data, a total of 13 categories were revealed. Three of these overlapped across the three groups of participants and one of these overlapped across two groups. Insight was mentioned only by the DPs. This therefore resulted in a total of five unique categories. These related to insight, context, extrafacial information, group and social context (including media), and alternative explanations. The latter four specifically relate to symptoms of DP and have enabled us to thoroughly explore and identify hallmark symptoms of the condition across childhood and adulthood. The most frequent codes from these categories (i.e. the symptoms which the participants discussed in the questionnaires and interviews most often) are considered hallmark symptoms of the condition and are presented in Table 8.

**Table 8:** The Hallmark Symptoms of DP in Adulthood and Childhood.

Hallmark Symptom
Confusion regarding the characters when watching films, TV and/or plays
Inability to identify people in photographs (including famous people, a personally familiar person, or oneself)
Teachers and/or guardians suggest screening for an alternative developmental disorder, yet this seems inappropriate
Appearing “lost” in a crowded place/large gathering (e.g. in the playground, at a train station)
Severity of difficulties increases in groups where everyone shares a characteristic (e.g. same uniform, same age or gender)
When in conversations, asking generic questions and nothing personal until a clue to their identity is given
Consistently avoids using other people’s names
Never introduces themselves to someone else, or two people to each other
Relying on extrafacial information to identify someone and failing to recognise someone when this changes or is unavailable (e.g. hair style or colour; voice or accent; gait or walk; clothing style or uniform)
Describing people by using extrafacial information (e.g. “that’s Mr X with the motorbike helmet”)
Confusing individuals who have extrafacial features in common, but are facially dissimilar
An inability to identify an unexpectedly encountered familiar person
Walking past and accidentally ignoring familiar people when in public (i.e. that individual is out of context)
A relative ease in recognising people when they appear in expected contexts (e.g. a work colleague in the workplace)
Believing others to be extraordinarily good at face recognition and/or being amazed by others’ abilities^a^
Struggling to reconstruct or imagine a person’s face in one’s mind and/or being unable to describe that face

^a^A symptom which is a personal belief, and not necessarily an outward behavioural characteristic. Thus, this symptom is more likely to be identified in oneself rather than in another person.

## Discussion

This study used qualitative methods to investigate DP – specifically, examining when individuals gained insight into their condition, and identifying common symptoms or characteristics that could be useful in the detection of DP. Individuals with DP, their unaffected SOs, and parents of children with the condition completed questionnaires and follow-up semi-structured interviews about their experiences of the condition. Data suggest that DPs have limited insight into their difficulties and several factors impact the likelihood of, and age at which, insight is gained. Questionnaires and interviews explored the age at which the condition can potentially be detected. In short, it appears that most individuals do not become aware of their own difficulties until adulthood. However, symptoms of DP may still be apparent in childhood, even if the affected individual is unaware of their difficulties at the time. This finding raises the possibility that the condition can be spotted by others, if provided with an appropriate symptom checklist. Thus, to aid the detection of DP in both adulthood and childhood, this paper lists sixteen evidence-based hallmark symptoms of the condition (see Table 8). Four categories relating directly to potential characteristics of the condition were revealed consistently across all three groups of participants. Yet, DP can only be detected if people are aware of the condition.

### Subjective Measures of Face Recognition Skills

Previous research proposes that individuals have a limited insight into their own face recognition abilities^36–38^ and it remains unclear whether those with DP have a greater level of insight into their abilities than typically developing individuals^52^. The question of whether individuals with DP are aware of their difficulties has important implications for identification of the disorder, especially given the fact that public awareness of DP is relatively low and most individuals only receive a diagnosis through self-referral to university laboratories. In line with this, 16 of the 50 DPs surveyed in this study believe that those living with the condition have a limited insight into their difficulties and, in turn, this means that some individuals living with the condition do not self-refer themselves to researchers (the category “Insight and Implications for Self-Referral”). However, the data also revealed several factors that might promote the development of insight in individuals with DP: of the 16 individuals who discussed problems with self-referring, 15 suggested that insight into the extent of their difficulties was only gained when they became aware that DP was a recognised condition. Six described that insight was only gained when they had a point of comparison and/or observed someone else’s ‘superior’ abilities. For example, one DP described how they only gained insight into the extent of their difficulties when a friend identified someone but they themselves were unable to recognise them, even after being told who they were. Therefore, it appears that awareness of DP and having the ability to compare one’s own face recognition skills to another person’s strongly impacts the likelihood of gaining insight into one’s own difficulties.

Another implication for the ability to detect DP, either in oneself or in another, is the severity of one’s difficulties. As the disorder is highly heterogeneous and varies in severity (e.g. refs^9,43,53^), it is likely that a more pervasive and serious symptomatology would allow insight to be gained at an earlier age, and for another person to more rapidly detect the difficulties. In line with this, four of our DPs specifically suggested that the gaining of insight depends upon the severity of the disorder. Taken together, this evidence suggests that a number of factors will influence the ability to detect DP in oneself, and consequently, self-report is not necessarily a reliable measure of face recognition difficulties. Identifying the condition, therefore, may fall to another person, and the use of a checklist which includes items derived from DPs, SOs and parents, may overcome the limitations of self-report.

### Age of Potential Detection

The present results suggest that individuals with DP appear to have a limited insight into their own difficulties until adulthood: only 12 DPs believed that they gained insight into the extent of their difficulties before reaching early adulthood (20 years of age). However, when asked in hindsight, over half of the DPs stated categorically that they have had difficulties since childhood. In addition, 42 DPs offered specific descriptions of their earliest experiences demonstrating their difficulties, and 26 of these occurred in childhood (i.e. before the age of 11 years). Furthermore, all three parents recognised their child’s DP when their child was aged 4 or 5 years old. In sum, the current data suggest that symptoms of DP can be present in childhood and in some cases, as reported by four of the adult DPs (see Table 7), can be potentially identified by another person, as young as 2–3 years of age.

The fact that DP may be able to be detected early in life is important for a number of reasons. First, children with DP risk a misdiagnosis of an alternative developmental disorder^41,42^. In our current sample, all three parents noted that teachers and/or caregivers had suspected their child of having a different condition than DP. Furthermore, adult DPs also reported that they and other people had attributed their face recognition difficulties to something else, including generalised memory problems, aging, laziness, or simply being absent minded. Identifying DP earlier in life would result in fewer misdiagnoses and accordingly, individuals would be more likely to receive suitable intervention and support. Parental responses indicated that early identification and support can have a substantial positive effect for children with DP: one of the parents spoke about the fact that the school their child attends is aware of their DP (and consequently are not attributing their difficulties to any other condition). Because of this, the child is coping well at school and the transitioning through school years has been comfortable for the child because of the support provided. Such an example suggests a second important implication for early detection, namely, that detecting DP sooner in some cases would decrease negative psychosocial consequences associated with the condition (e.g. ref.^15^). The psychosocial consequences of DP were not the focus of the current study. However, the impact DP has on one’s behaviour in social contexts was consistently revealed as a category across all three groups of participants, highlighting not only its role as an indicator of DP, but also the importance of considering the impact of DP on individuals’ wellbeing. Responses from adult DPs and parents highlight a striking association between misdiagnosis or misattribution of difficulties and negative psychosocial consequences. In sum, detecting DP and receiving a correct diagnosis and appropriate support could mitigate potential negative effects on the individual’s psychosocial wellbeing.

Furthermore, identifying DP (especially in childhood) could reduce the chances of individuals being placed in potentially dangerous situations (e.g. ref.^42^). Four of the adult DPs discussed experiences which could have been extremely dangerous, including accepting lifts with “strangers” and walking out of shops with a stranger believing that person to be a relative. Increasing awareness and detecting DP earlier will certainly help combat these potentially dangerous instances: this is supported by the fact that none of the parents discussed such experiences, presumably because they are aware of such potentially dangerous situations and have taken steps to limit them. Finally, it would be extremely beneficial for research purposes if we were better able to detect DP in both adulthood and childhood. Detecting DP more easily and earlier in life would increase the number of individuals being referred to researchers and, subsequently, increase opportunities for future research. In addition, the benefits of training and the success of remedial techniques appear to be more effective when administered earlier in life (see ref.^54^ for a review), and thus, earlier detection of DP would be more beneficial in order to help those living with the condition. All things considered, detecting DP in both children and adults, and as soon as possible, is essential.

### Hallmark Symptoms

The present study sought to identify and present the first evidence-based checklist to aid the identification of DP. To date, the symptoms that have been presented for DP have been drawn from informal discussions with those living with the condition and/or the available literature (e.g. ref.^30^). However, such discussions with DPs have not been documented or subjected to any formal analysis (but see refs^15,16^). Across all data, a total of 13 categories were revealed. After combining or collapsing the categories that appeared in multiple groups of participants, a total of five different categories remained, four of which specifically related to symptoms of DP. In order of the total percentages of individual mentions, these were those relating to group and social contexts (including media), extrafacial information, the importance of context, and alternative explanations, and have enabled us to thoroughly explore and identify hallmark symptoms of the condition (Table 8).

Whilst we present sixteen hallmark symptoms of DP, we must highlight that some of the symptoms included in the checklist may be more suitable to identify DP in oneself than in another person. Many individuals with DP develop ways in which to cope in their daily lives^33^ and can conceal their difficulties from others. Thus, there is the potential that some of the symptoms describe characteristics which DPs are aware they are doing, and unaffected individuals do not notice is happening. For example, the symptom “consistently avoids using other people’s names” could be something which may go unnoticed by another person; it is not unusual to use terms of endearment or to be called a term of endearment, but it is unusual to use terms of endearment to hide the fact you cannot recognise who you are talking to. Thus, this may be a characteristic which will be noticed more in oneself, than from another person’s point of view. Another symptom presented in the checklist is “believing others to be extraordinarily good at face recognition and/or being amazed by others’ abilities”. This is a personal belief rather than an outward characteristic, and another adult may be unable to answer this question without questioning that individual. In sum, a variety of factors, including the development of coping mechanisms and personal understanding and beliefs, means that there may be a small number of symptoms on the checklist which are more able to identify DP in oneself rather than in another person.

Furthermore, there may be some symptoms which identify DP in children more accurately than in adults, or vice versa. A category relating to alternative explanations for one’s face recognition difficulties and misdiagnosis was discussed by both the DPs and the parents. Based upon these data, the symptom “teachers and/or guardians suggest screening for an alternative developmental disorder, yet this seems inappropriate” was presented as one of the sixteen hallmark symptoms. This symptom was derived from the present parental data, so it would not be surprising if this detects DP in childhood more so than in adulthood. Nevertheless, the awareness and subsequent prevalence rates of alternative developmental disorders, such as ASD, have gradually increased through the 1990s and through the 21^st^ century^55^, and the prevalence rates of ASD in adulthood mirror those reported in childhood (e.g. ref.^56^). Consequently, is not implausible that this symptom may be seen in the adult population and, accordingly, this remains as one of the sixteen symptoms.

In sum, Table 8 presents the first symptom checklist which aims to aid the detection of DP in oneself and in another person, in both adulthood and childhood. It is fundamental to point out that the checklist was devised by data obtained from participants who have confirmed DP, are SOs of someone with confirmed DP, or are parents of DP subjects. It would therefore be valuable to assess how well this checklist differentiates those who have met the criteria for DP, and those who self-report face recognition difficulties but do not meet the current diagnostic criteria for the condition. Furthermore, a large, new sample of DPs, SOs and parents are needed in order to fully validate the checklist and determine which questions should receive a higher weighting in a self-report version of the questionnaire, or when working with children with suspected DP. In sum, these symptoms are therefore not presented as a diagnostic measure, but to help identify hallmark DP characteristics so that individuals can be referred to the correct professionals for a suitable screening session.

## Conclusions

The present study reports that individuals with DP have limited insight into their own abilities, suggesting that its detection may fall to unaffected others. Furthermore, the age at which DPs become aware of their own difficulties (i.e. they understand that they have a deficit relative to others) varies widely, and the vast majority of DPs only become aware of their difficulties in adulthood. However, DPs’ reflections on past experiences, together with parental responses, indicate that the condition can be detected in children as young as 2–3 years of age. To aid the detection of DP, we present the first list of everyday symptoms which can be used to assist the detection of the condition in both adults and children, and in oneself and in another person.

## Electronic supplementary material

Supplementary Information
