# Supplementary material for: Identifying Hallmark Symptoms of Developmental Prosopagnosia for Non-Experts
Source: Sci Rep. 2018 Jan 26;8:1690. doi: 10.1038/s41598-018-20089-7 (PMC5786061; doi:10.1038/s41598-018-20089-7)
Supplement: Supplementary file 1 — Supplementary Information [file 41598_2018_20089_MOESM1_ESM.pdf]

Identifying Hallmark Symptoms of Developmental Prosopagnosia for Non-Experts

Ebony Murray[1*], Peter J. Hills[1], Rachel J. Bennetts[2,] Sarah Bate[1],

[1] Department of Psychology, Bournemouth University, Bournemouth, Dorset, UK

[2] Department of Biological and Experimental Psychology, Queen Mary University of London, London, UK

* Corresponding author

E-mail: emurray@bournemouth.ac.uk (EM)

Supplementary Information:

Supplementary Table 1. The Demographics and Scores on the Diagnostic Measures for Adult DP Participants.

Supplementary Table 2. The Demographics and Scores on the Diagnostic Measures for the Children.

Supplementary Table 3. The Demographics of the SO Participants.

Supplementary Methods 4. Questionnaire Questions Completed by DP Participants.

Supplementary Methods 5. Questionnaire Questions Completed by SO Participants.

Supplementary Methods 6. Questionnaire Questions Completed by Parents.

Supplementary Methods 7. The Main Interview Questions for all Participants.

Supplementary Table 1. The Demographics and Scores on the Diagnostic Measures for Adult DP Participants.

Participant Code	Age at time of testing	CFMT score	CFPT (upright)	Famous faces %
DPM63*	63	29	54	23.64
DPF49*	49	35	66	18.75
DPF77*	77	39	70	35.56
DPF56*	56	42	48	37.74
DPF68*	68	38	60	56.00
DPM65*	65	37	58	50.00
DPM53*	53	33	54	31.03
DPM48*	48	38	72	61.67
DPM68*	68	28	52	43.40
DPF49*	49	32	80	32.14
DPF29*	29	40	52	47.37
DPF70*	70	38	58	34.62
DPF32	32	33	48	38.89
DPF69*	69	39	64	52.54
DPF37*	37	42	78	33.90
DPM70*	70	37	84	44.00
DPF53*	53	42	96	54.50
DPF52*	52	34	78	71.90
DPF57	57	30	64	N/A
DPM51*	51	41	80	75.40
DPF29*	29	30	74	58.00
DPF37	37	35	80	N/A
DPF46*	46	39	80	67.30
DPM40*	40	36	80	77.60
DPM48	48	39	90	N/A
DPF54*	54	35	88	N/A
DPF44*	44	39	80	39.00
DPF39	39	35	78	N/A
DPF25	25	32	66	N/A
DPM40	40	42	70	N/A
DPF64	64	35	82	94.00
DPF56	56	38	60	61.11
DPF62	62	39	90	N/A
DPM53	53	41	46	52.80
DPF43	43	41	58	N/A
DPF54	54	30	58	72.80
DPM52	52	39	96	70.00
DPF52	52	35	94	N/A
DPF48	48	38	36	60.00
DPF69	69	48	72	51.00
DPF60	60	26	84	N/A
DPM68	68	38	90	N/A
DPM76	76	29	58	49.00
DPM72	72	37	74	N/A
DPM62	62	32	80	N/A
DPM58	58	38	80	N/A

DPF52	52	31	82	N/A
DPF27	27	37	43	45.95
DPF53	53	35	88	N/A
DPF51	51	39	84	N/A

** Participant also completed the interview stage of this study; N/A indicates that that participant did not complete this task.*

<u>Supplementary Table 2.</u> The Demographics and Scores on the Diagnostic Measures for the Children.

Participant Code	Age at time of testing	Face memory %	Bike memory %	Face matching %	Bike matching %
PM6	6	27.08	35.41	33.33	53.33
PM5*	5	39.58	68.75	86.66	80.00
Participant Code	Age at time of testing	CFMT score	CFPT score		
PM15*	15	38	72		

** Participant also completed the interview stage of this study. Participant PM15 was screened as a teenager and therefore completed the CFMT and CFPT to determine DP.*

<u>Supplementary Table 3.</u> The Demographics of the SO Participants

Participant Code	Age at time of testing
SOF62*	62
SOF43*	43
SOF65*	65
SOF51*	51
SOF71*	71
SOM32	32
SOM72*	72
SOM35	35
SOF64*	64
SOF74*	72
SOM54	54
SOF19	19
SOM38	38
SOM56	56
SOF42	42
SOF46	46
SOM56	56
SOM46	46
SOM63	63
SOF44	44
SOM54	54
SOM53	53
SOF64	64
SOF58	58
SOM53	53
SOM54	54

** Participant also completed the interview stage of this study*

Supplementary Methods 4. Questionnaire Questions Completed by DP Participants.

1. When did you first participate in a screening session which the research team at Bournemouth University carried out?
    a. Have you participated in any other research projects, either with Bournemouth University or elsewhere (e.g. eye-tracking studies, training studies)?

2. Do you believe you have had difficulty recognising faces since childhood?
    a. If so, looking back, are there any experiences which you think could have been signs that you have difficulty with face recognition (e.g. choosing friends in the classroom with particular characteristics so they 'stood out')?

3. What is your earliest memory/experience which illustrates your difficulties recognising faces? How old were you at this time?

4. Are there any settings where your face recognition difficulties are more prominent (e.g. in the workplace where similar clothing is worn by a number of people)?

5. Do you find you have the same level of difficulty recognising everybody, or are certain people easier to recognise than others?

    a. If some people are easier to recognise than others, please detail this further below.

6. Reflecting on your own experience, please list any hallmark indicators you feel there may be of prosopagnosia.

7. Do you believe that people with prosopagnosia have insight into the severity of their face recognition difficulties? Is there an age at which this becomes more accurate?

Supplementary Methods 5. Questionnaire Questions Completed by SO Participants.

1. Please state your relationship to the person who has asked you to complete this questionnaire (e.g., partner, sibling, friend).
    a. How long have you known this person?
    b. How often do you see or speak to this person on average (e.g., daily, weekly, monthly)?

2. How long have you known your significant other struggles with face recognition?
    a. Did you realise that your significant other had trouble with faces without them having to tell you?
    b. Looking back, are there any experiences which you think could have been signs that he/she had difficulty with face recognition (e.g. mistaking people who you could easily tell apart)?

3. What is the earliest memory/experience which would illustrate your significant other's difficulties with face recognition? How old was your significant other at the time?

4. Are there any settings where you think your significant other's face recognition difficulties are more prominent (e.g. in the workplace if similar clothing is worn by a number of people, at large family gatherings)?

5. Do you believe your significant other has the same level of difficulty recognising everybody, or are certain people easier or more difficult to recognise than others?

   a. If some people appear to be easier or more difficult to recognise than others, please detail this further below.

6. Reflecting on your own experiences, please list any hallmark indicators you feel there may be of prosopagnosia.

Supplementary Methods 6. Questionnaire Questions Completed by Parents.

1. When did your child first participate (or when are they due to participate) in a screening session which the research team at Bournemouth University carried out?
   a. Has your child participated in any other research projects studying face recognition problems, either with Bournemouth University or elsewhere (e.g. assessments, eye-tracking studies, training studies)?

2. How long have you known your child struggles with face recognition?
   a. Looking back, are there any experiences which you think could have been signs that he/she had difficulty with face recognition (e.g. mistaking people who you could easily tell apart)?

3. What is the earliest memory/experience which would illustrate your child's difficulties with face recognition?
   a. How old was your child at the time?

4. Are there any settings where you think your child's face recognition difficulties are more prominent (e.g. at school if there are uniforms or other dress codes, at large family gatherings)?

5. Do you believe your child has the same level of difficulty recognising everybody, or are certain people easier or more difficult to recognise than others?
   a. If some people appear to be easier or more difficult to recognise than others, please detail this further below.

6. Reflecting on your own experiences, please list any hallmark indicators you feel there may be of prosopagnosia in children.

Supplementary Methods 7. The Main Interview Questions for all Participants.

Adult DPs

1. Did you ever believe that faces were uninformative cues to identity for everyone? At what age did this change?

2. Did you have a specific experience which particularly highlighted your difficulties, or was it a combination of past experiences?
   a. If there was a specific event or experience, please tell me about it.

3. Do you find your face recognition difficulties cause you to do certain things or act in certain ways when you're around people? If so, please detail/give example(s) where you can.

SOs

1. Can you think of any examples/experiences which illustrate your significant other's difficulties recognising faces?

2. Do you think your significant other's face recognition difficulties cause him/her to do certain things or act in certain ways when he/she is around people? If so, please provide details/give example(s) where possible.
    a. Do you think having face recognition difficulties has an impact on your significant other's social interactions? If so, are there any social interactions/groups of people who are most affected?

3. Do you think that your significant other's face recognition difficulties cause him/her to approach situations or activities in a particular way? If so, please provide details/give example(s) where possible.
    a. Do you think having face recognition difficulties prevents your significant other from carrying out certain everyday tasks? If so, which tasks?

Parents

1. Can you think of any examples/experiences which illustrate your child's difficulties recognising faces?

2. Do you think your child's face recognition difficulties cause him/her to do certain things or act in certain ways when he/she is around people? If so, please provide details/give example(s) where possible.
    a. Do you think having face recognition difficulties has an impact on your child's social interactions? If so, are there any social interactions/groups of people who are most affected?

3. Do you think that your child's face recognition difficulties cause him/her to approach situations or activities in a particular way? If so, please provide details/give example(s) where possible.
    a. Do you think having face recognition difficulties prevents your child from carrying out certain everyday tasks? If so, which tasks?
    b. Are there any situations or tasks that you try to keep your child away from due to their prosopagnosia? If so, why?

4. Have people ever tried to attribute your child's face recognition difficulties to something else (e.g., a personality type, another condition, more general problems with memory and attention)? If so, please provide details/give examples where possible.

5. Have you ever had contact with a medical/educational/occupational professional about your child's prosopagnosia? Was this helpful?
